# Diagnosis of joint invasion in patients with malignant bone tumors: value and reproducibility of direct and indirect signs on MR imaging

**DOI:** 10.1007/s00330-022-08586-w

**Published:** 2022-03-08

**Authors:** Jannis Bodden, Jan Neumann, Michael Rasper, Alexander A. Fingerle, Carolin Knebel, Rüdiger von Eisenhart-Rothe, Katja Specht, Carolin Mogler, Christine Bollwein, Benedikt J. Schwaiger, Alexandra S. Gersing, Klaus Woertler

**Affiliations:** 1 Department of Diagnostic and Interventional Radiology, Technical University of Munich, Ismaninger Str. 22, 81675 Munich, Germany; 2 Department of Radiology and Biomedical Imaging, University of California, San Francisco, 185 Berry St, Lobby 6, Suite 350, San Francisco, CA 94107 USA; 3 Department of Radiology, Kantonsspital Muensterlingen, Spitalcampus 1, 8596, Muensterlingen, Switzerland; 4 Department of Orthopaedic Surgery, Technical University of Munich, Ismaninger Str. 22, 81675 Munich, Germany; 5 Interdisciplinary Musculoskeletal Tumor Center, Technical University of Munich, Ismaninger Str. 22, 81675 Munich, Germany; 6 Institute of Pathology, Technical University of Munich, Ismaninger Str. 22, 81675 Munich, Germany; 7 Department of Diagnostic and Interventional Neuroradiology, Technical University of Munich, Ismaninger Str. 22, 81675 Munich, Germany; 8 Department of Neuroradiology, University Hospital, LMU Munich, 81377 Munich, Germany

**Keywords:** Bone neoplasms, Neoplasm staging, Osteosarcoma, Chondrosarcoma, Magnetic resonance imaging

## Abstract

**Objectives:**

To evaluate the performance and reproducibility of MR imaging features in the diagnosis of joint invasion (JI) by malignant bone tumors.

**Methods:**

MR images of patients with and without JI (*n *= 24 each), who underwent surgical resection at our institution, were read by three radiologists. Direct (intrasynovial tumor tissue (ITT), intraarticular destruction of cartilage/bone, invasion of capsular/ligamentous insertions) and indirect (tumor size, signal alterations of epiphyseal/transarticular bone (bone marrow replacement/edema-like), synovial contrast enhancement, joint effusion) signs of JI were assessed. Odds ratios, sensitivity, specificity, PPV, NPV, and reproducibilities (Cohen’s and Fleiss’ *κ*) were calculated for each feature. Moreover, the diagnostic performance of combinations of direct features was assessed.

**Results:**

Forty-eight patients (28.7 ± 21.4 years, 26 men) were evaluated. All readers reliably assessed the presence of JI (sensitivity = 92–100 %; specificity = 88–100%, respectively). Best predictors for JI were direct visualization of ITT (OR = 186–229, *p * < 0.001) and destruction of intraarticular bone (69–324, *p *< 0.001). Direct visualization of ITT was also highly reliable in assessing JI (sensitivity, specificity, PPV, NPV = 92–100 %), with excellent reproducibility (*κ* = 0.83). Epiphyseal bone marrow replacement and synovial contrast enhancement were the most sensitive indirect signs, but lacked specificity (29–54%). By combining direct signs with high specificity, sensitivity was increased (96 %) and specificity (100 %) was maintained.

**Conclusion:**

JI by malignant bone tumors can reliably be assessed on preoperative MR images with high sensitivity, specificity, and reproducibility. Particularly direct visualization of ITT, destruction of intraarticular bone, and a combination of highly specific direct signs were valuable, while indirect signs were less predictive and specific.

**Key Points:**

*• Direct visualization of intrasynovial tumor was the single most sensitive and specific (92–100%) MR imaging sign of joint invasion.*

*• Indirect signs of joint invasion, such as joint effusion or synovial enhancement, were less sensitive and specific compared to direct signs.*

*• A combination of the most specific direct signs of joint invasion showed best results with perfect specificity and PPV (both 100%) and excellent sensitivity and NPV (both 96 %).*

**Supplementary Information:**

The online version contains supplementary material available at 10.1007/s00330-022-08586-w.

## Introduction

Malignant bone tumors account for approximately 6% of all cancer cases under the age of 20 years [1, 2]. The most common types of malignant bone neoplasms affecting young patients are osteosarcomas and Ewing sarcomas, with relative frequencies of 50 % and 40 %, respectively. Chondrosarcomas can occur at any age, but 70 % of the affected patients are older than 40 years. While radical resection and amputation were the only available treatment options for these tumors until the 1970s, multimodal therapy, including (neo-)adjuvant chemotherapy, radiation therapy, and surgery, has become the reference standard today [3]. However, neoadjuvant chemotherapy has been found not to impact imaging results or resectability in a favorable manner and resection of the adjacent joint remains inevitable in most cases [1, 4]. The surgical approach is determined by the joint invasion (JI) status: whereas unaffected joints may be resected intraarticularly, the presence of JI necessitates extra-articular resection to prevent local recurrence, which is associated with a poor prognosis [5, 6]. However, extra-articular resection is technically demanding and may lead to unfavorable functional results [7, 8].

Magnetic resonance (MR) imaging is the standard imaging method for the local staging of malignant bone tumors [9, 10]. Providing excellent soft tissue contrast and three-dimensional anatomic information, extraosseous tumor growth and the proximity to critical structures, like vessels or nerves, can be assessed precisely [11]. Previous studies have also investigated the feasibility of JI assessment on MR images [10, 12–14]. Schima et al investigated epiphyseal involvement, presence of intrasynovial tumor tissue, invasion of the joint capsule, intraarticular destruction of cortical bone or cartilage, invasion of the cruciate ligaments, and joint effusion as MR criteria for tumor invasion of the knee joint [12]. Ozaki et al defined cartilage disruption, acetabular/epiphyseal signal alterations, and marked joint effusion as signs of JI at the hip [13]. However, information on the performance of these imaging features is scarce and reported specificities for MR-based JI assessment differ dramatically [10, 12, 13]. Moreover, previous studies did not analyze the reproducibility of the individual features assessed on MR imaging.

The aim of this study was thus to evaluate the performance and reproducibility of previously described and new MR imaging features in the diagnosis of JI in patients with malignant bone tumors.

## Materials and methods

### Cohort

Approval of the Institutional Review Board has been obtained prior to this study and written informed consent was waived. Pre-treatment MR examinations of patients with malignant bone neoplasms, who had undergone joint resection at our musculoskeletal tumor center between 01/2009 and 12/2019, were obtained from the imaging database. Examinations obtained after chemotherapy were not included for two reasons: First, apparent tumor size reduction under neoadjuvant treatment should not be mistaken as reversal of JI, as intraarticular spread may already have occurred. The planning of tumor resection is therefore almost always based on preoperative images with regard to the initial tumor extent. Second, neoadjuvant treatment may induce signal alterations that may be mistaken for tumor tissue and thus be subject to false-positive assessments [4]. To limit the variability of MR protocols, included exams were required to be performed for primary bone tumor screening purposes. Tumor joint adjacency was defined as tumor invasion of either the metaphysis of the joint-forming long bone, the acetabulum, or the glenoid. Cases not presenting one of these features were excluded. Availability of pre-therapeutic scans was obligatory, as imaging features have been shown to be influenced by neoadjuvant therapy [15–17]. Definitive statements on JI status were obtained from pathologic reports of the local Institute of Pathology. Specimens with unclear reports were reviewed by two pathologists (K.S. and C.B.) and JI status was determined.

### Image acquisition and evaluation

MR images were acquired in at least two planes: short tau inversion recovery/fat-saturated intermediate-weighted and pre- and post-contrast T_1_-weighted sequences were oriented along the longitudinal axis of the articulating long bone(s), whereas T_2_-weighed and fat-saturated post-contrast T_1_-weighed sequences were oriented along the short axis. Detailed information on sequence parameters is available as supplementary material. Images were independently reviewed by three radiologists (K.W., A.A.F., and M.R., with 26, 10, and 8 years of experience in musculoskeletal radiology, respectively) using picture archiving and communication system (PACS) workstations. Readers were blinded regarding clinical information, surgical outcomes, and histopathological findings. Images were evaluated using a standardized scoring sheet, assessing the following indirect (1.–4.) and direct (5.–7.) signs of JI:

1. Epiphyseal/Acetabular signal abnormalities:

- 1.1 Bone marrow replacement (iso-/hypointense to muscle tissue on T_1_-weighted images)
- 1.2 Edema-like signal intensity (hyperintense to muscle on T_1_-weighted images and markedly hyperintense on STIR-/intermediate-weighted/T_2_-weighted images).
  2. Signal abnormalities affecting the transarticular bone (transarticular to primary tumor site):

- 2.1 Bone marrow replacement (see 1.1.)
- 2.2 Edema-like signal intensity (see 1.2.)
  3. Synovial contrast enhancement
  4. Joint effusion
  5. Direct visualization of ITT (tumor tissue definitively crossing the synovial membrane toward the joint space, having direct contact to synovial fluid)
  6. Intraarticular destruction of:
- 6.1 Bone (discontinuity of intraarticular cortical bone)
- 6.2 Cartilage (discontinuity of articular cartilage)
  7. Invasion of:
- 7.1 Cruciate ligament(s) (knee joint)
- 7.2 Ligamentum teres (hip joint)
- 7.3 Capsular insertions (all joints)

Readers further measured the largest tumor diameter in centimeter and tumors were divided into two groups (larger than average, smaller than average), for each reader. The overall imaging diagnoses (JI: yes/no), and the subjective certainty of each reader’s diagnosis (5-point Likert-scale, 1: definitely present; 2: likely present; 3: uncertain; 4: likely not present; 5: definitely not present), were documented. Image quality was assessed by one reader using a 4-point ordinal scale (poor, moderate, good, excellent). Reader 1 also documented the MR sequence and image orientation most relevant for the diagnosis. Readings were repeated for intra-reader reproducibility measurements (K.W., 90 days between readings).

### Combinations of imaging features

Following the primary analysis of individual imaging features, three dummy variables were created for Reader 1, based on the following criteria: MaxSens was defined as positive if at least one of all direct imaging features with a good inter-reader reproducibility (Fleiss’ *κ* ≥ 0.6) and a good sensitivity (≥ 80 %, in each reader) was achieved. MaxSens was negative if all of the variable-defining imaging features were negative. MaxSpec was positive if at least one direct imaging feature with a good inter-reader reproducibility (Fleiss’ *κ* ≥ 0.6) and a good specificity (≥ 80 %, in each reader) was positive, and negative if all of the defined imaging features were negative. AllDirect was positive if at least one direct sign of JI was positive, and negative if all direct signs of JI were negative.

### Statistical analysis

Data analysis was performed by B.J.S. and J.B. using STATA version 15 software (StataCorp LP). All statistical tests were performed with a two-sided level of significance (*α*) of 0.05. Inter-group differences in demographics were compared using Pearson’s chi-square test for categorical data (gender, side, site, tumor type) and an independent samples *t* test for continuous data (age at diagnosis). In a primary analysis, exact logistic regression models were used to determine associations between the observation of individual imaging features and JI status. Moreover, sensitivity, specificity, positive predictive value (PPV), and negative predictive value (NPV) were calculated using crosstabs for each imaging feature and each reader, respectively. Infinity as upper limit of the 95% CI was noted as “+inf.”

In the secondary analysis, sensitivity, specificity, PPV, and NPV of the aforementioned dummy variables (MaxSens, MaxSpec, and AllDirect) were calculated.

Fleiss’ *κ* was used to determine inter-reader reliability for repeated image analysis in each imaging feature between readers. Intra-reader reproducibility for repeated measures was calculated using Cohen’s *κ*.

## Results

### Cohort characteristics

**Fig. 1:**
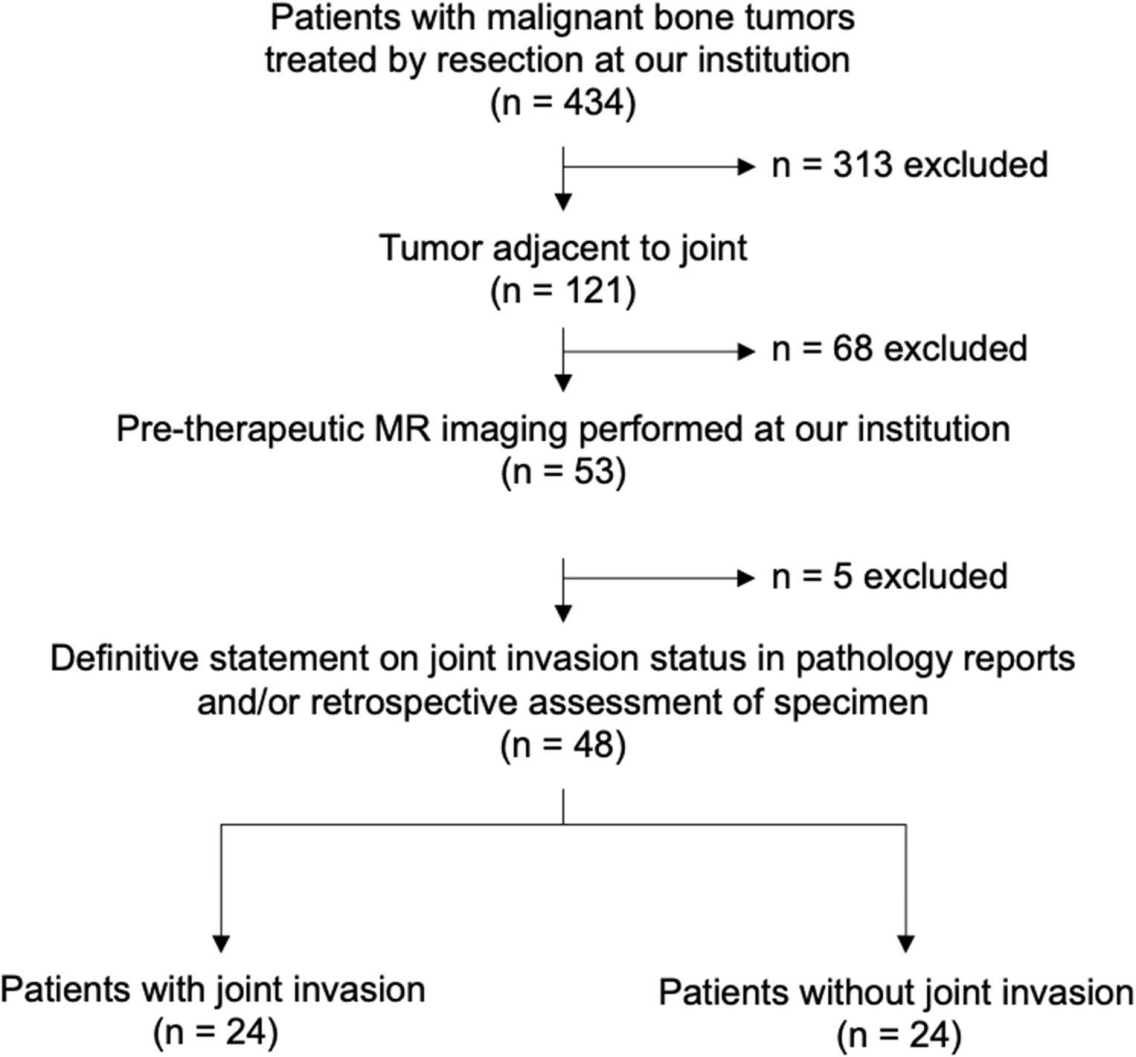
Flow diagram of study population and patient recruitment

**Fig. 2:**
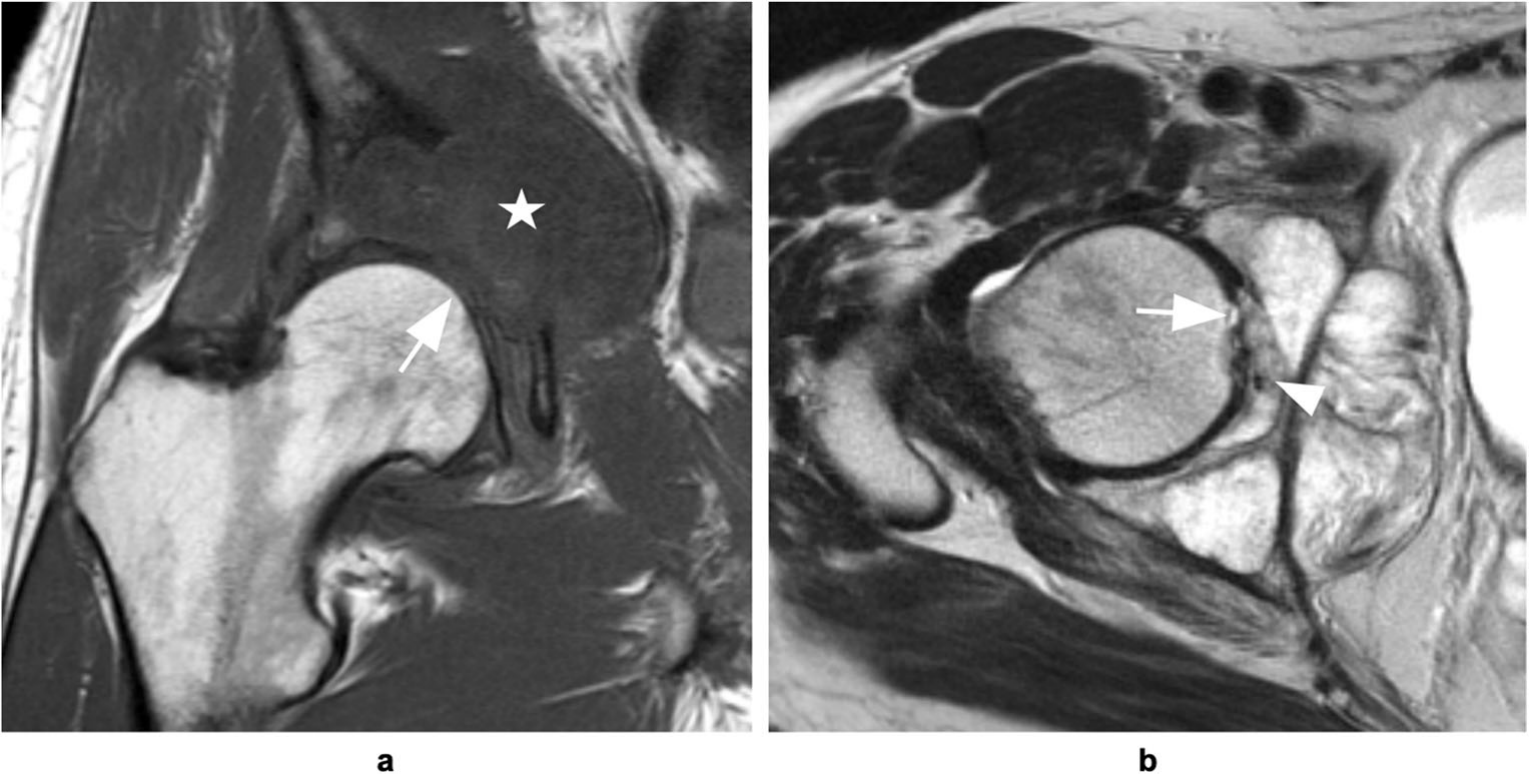
Chondrosarcoma of the pelvis invading the right hip joint. **a** Coronal T_1_-weighted TSE image demonstrates bone marrow replacement at the acetabulum and destruction of intraarticular bone (*arrow*) caused by bone tumor extending into the pelvis (*star*). **b** Axial T_2_-weighted TSE image shows tumor invasion of the ligamentum teres (*arrow*) as well as direct visualization of intraarticular tumor tissue in the acetabular fossa (*arrowhead*). Note the absence of relevant joint effusion and typical lobulated appearance and high signal intensity of cartilaginous mass

Of 434 patients with malignant bone tumors treated at our institution, 313 were excluded as metaphyseal invasion was absent and/or patients did not undergo resection (Figure 1). Further 68 patients were excluded, because pre-therapeutic MR imaging was unavailable, and five patients were excluded, because the pathologic report lacked a definitive statement on JI and retrospective assessment of the specimens was impossible. We finally included 48 patients (age in years ± SD: 28.7 ± 21.4 years; sex: 26 men, 22 women) with joint adjacent malignant bone tumors in this study (Table 1). JI was more common in older patients and men were affected more frequently (age, no JI: 16.5 ± 12.0 vs. JI 40.8 ± 22.5, *p* < 0.001; sex, no JI: 14 women, 10 men; JI: 8 women, 16 men, *p* = 0.08). Most tumors were located at the knee (*n *= 28), exceeding both tumors at hips (*n *= 13) and shoulders (*n* = 7) combined. However, histopathologic findings revealed a higher prevalence of JI in hips (Figure 2) (11/13, 85 %) compared to knees (10/28, 36 %) and shoulders (3/7, 43 %) (*p* = 0.006). Tumor entities comprised osteosarcomas (*n* = 30), chondrosarcomas (*n* = 11), Ewing sarcomas (*n* = 4), two secondary malignancies, and one pleomorphic sarcoma. JI was most frequently diagnosed in chondrosarcomas, secondary malignancies, and pleomorphic sarcoma (osteosarcoma 43%; chondrosarcoma 73 %; Ewing sarcoma 0 %; others 100 %, *p* = 0.006).

**Table 1:** Cohort demographics

Parameter		Without joint invasion (*n* = 24)	With joint invasion (*n* = 24)	Total (*n* = 24)
Age, mean ± Standard deviation (years)		16.5 ± 12.0	40.8 ± 22.5	28.7 ± 21.4
Sex, *n* (%)	Men	10/24 (42 %)	16/24 (67 %)	26/48 (54 %)
	Women	14/24 (58 %)	8/24 (33 %)	22/48 (46 %)
Tumor site, *n* (%)	Shoulder	4/24 (17 %)	3/24 (12 %)	7/48 (15 %)
	Hip	2/24 (8 %)	11/24 (46 %)	13/48 (27 %)
	Knee	18/24 (75 %)	10/24 (42 %)	28/48 (58 %)
Entity, *n* (%)	Osteosarcoma	17/24 (71 %)	13/24 (54 %)	30/48 (63 %)
	Chondrosarcoma	3/24 (12 %)	8/24 (33 %)	11/48 (23 %)
	Ewing sarcoma	4/24 (17 %)	0	4/48 (8 %)
	Pleomorphic sarcoma	0	1/24 (4 %)	1/48 (2 %)
	Secondary malignancy	0	2/24 (8 %)	2/48 (4 %)

### Image analysis

Reader 1 rated image quality as good or excellent in 42 of 48 cases and as moderate in the remaining six cases. T_2_-TSE was regarded as the most valuable sequence for determining JI status in most cases (35/48, 72.9%) and the conclusion certainty was “uncertain” in none of the cases. Concerning the overall imaging diagnoses, sensitivity and specificity were excellent in all readers (sensitivity: 92–100%; specificity: 88–100%) (Table 2).

**Table 2:** Diagnosis of Joint Invasion: Overall imaging diagnoses

Parameter	Reader 1			Reader 2			Reader 3		
	%		95% CI	%		95% CI	%		95% CI
Sensitivity	92	(22/24)	[73, 99]	100	(24/24)	[86, 100]	100	(24/24)	[86, 100]
Specificity	100	(24/24)	[86, 100]	88	(21/24)	[68, 97]	88	(21/24)	[68, 97]
PPV	100	(22/22)	[85, 100]	89	(24/27)	[74, 96]	89	(24/27)	[74, 96]
NPV	92	(24/26)	[76, 98]	100	(21/21)	[85, 100]	100	(21/21)	[85, 100]

*PPV*, positive predictive value; *NPV*, negative predictive value; *95% CI*, 95% Confidence interval

### Indirect signs of joint invasion

JI was strongly associated with epi-/apophyseal signal alterations reflecting cellular infiltration and findings were highly reproducible (odds ratio (OR) = 15.6–18.3, *p* = 0.002–0.004; Fleiss *κ* [95% CI] = 0.96 [0.80 – 1.00]; Table 3). This imaging feature was moreover found to be highly sensitive for JI and had a high NPV in each reader (sensitivity: 96%, NPV: 91–92%; Table 4). In comparison, edema-like signal alterations at the same location were of lesser diagnostic value (OR = 0.6–4.8, *p* = 0.03–0.56; sensitivity: 33–83%; NPV: 45–75%). Transarticular bone marrow signal alterations for both cellular infiltration and edema-like signal alterations had an excellent PPV (100%). However, both of these indirect signs were observed rarely, considerably affecting odds ratios and sensitivities (OR = 1.0–2.5, *p* = 0.49–1.00; sensitivity = 1 to 2/24, 4–8%). Joint effusion was associated with increased odds for JI and also showed a substantial inter-reader agreement, but was neither markedly sensitive nor specific and the NPV was limited (OR = 4.8–25.3, *p* = < 0.001–0.02; Fleiss *κ* = 0.70; sensitivity: 54–63%; specificity: 75–96%; NPV: 67–69%). Although odds for JI were comparably higher in cases with synovial contrast enhancement, reproducibility of this imaging feature was only moderate and its specificity limited (OR = 7.5 – 8.7, *p* = 0.007–0.06; Fleiss *κ* = 0.51; sensitivity: 87–96%; specificity: 29–54%). In our cohort, a maximum tumor diameter greater than 10.5 cm was not associated with increased odds for JI and this criterion was neither sensitive nor specific (*p* ≥ 0.56; sensitivity: 29–50%; specificity: 63–79%).

**Table 3:** Associations of imaging features and JI

Imaging feature		Reader	OR	95% CI	p*	Fleiss’ *κ*	95% CI	Cohen’s *κ*	95% CI
Tumor size > 10.5 cm		1	1.0	[0.3, 3.8]	1.00	0.67	[0.50, 0.83]	0.95	[0.86, 1.00]
		2	1.6	[0.4, 7.5]	0.74				
		3	1.7	[0.5, 6.2]	0.56				
Epiphyseal/acetabular	Bone marrow replacement	1	15.6	[1.9, 743]	**0.004**	0.96	[0.80, 1.00]	1.00	[1.00, 1.00]
		2	18.3	[2.2, 873]	**0.002**				
		3	18.3	[2.2, 873]	**0.002**				
	Edema-like signal intensity	1	0.6	[0.2, 2.2]	0.56	0.40	[0.24, 0.56]	0.52	[0.27, 0.77]
		2	2.7	[0.7, 12.3]	0.21				
		3	4.8	[1.1, 25.4]	**0.03**				
Transarticular	Bone marrow replacement	1	1.0	[0.3, + inf]	1.00	0.74	[0.58, 0.91]	1.00	[1.00, 1.00]
		2	1.0	[0.3, + inf]	1.00				
		3	2.5	[0.2, + inf]	0.49				
	Edema-like signal intensity	1	2.5	[0.2, + inf]	0.49	0.74	[0.58, 0.91]	1.00	[1.00, 1.00]
		2	1.0	[0.0, + inf]	1.00				
		3	1.0	[0.0, + inf]	1.00				
Synovial contrast enhancement		1	8.7	[1.0, 426]	0.06	0.51	[0.35, 0.68]	0.81	[0.55, 1.00]
		2	7.5	[1.6, 50.1]	**0.007**				
		3	7.5	[1.6, 50.1]	**0.007**				
Joint effusion		1	4.8	[1.3, 21.0]	**0.02**	0.70	[0.53, 0.86]	0.77	[0.58, 0.96]
		2	25.3	[3.1, 1204]	**< 0.001**				
		3	14.4	[2.6, 155]	**< 0.001**				
Direct visualization of ITT		1	229	[29.8, + inf]	**< 0.001**	0.83	[0.67, 1.00]	0.95	[0.86, 1.00]
		2	229	[29.8, + inf]	**< 0.001**				
		3	186	[17.8, 10848]	**< 0.001**				
Destruction of	Bone	1	324	[25.2, 22596]	**< 0.001**	0.72	[0.55, 0.88]	0.91	[0.79, 1.00]
		2	75.4	[8.7, 3780]	**< 0.001**				
		3	68.8	[10.0, + inf]	**< 0.001**				
	Cartilage	1	12.5	[1.7, + inf]	**0.009**	0.57	[0.40, 0.73]	0.85	[0.65, 1.00]
		2	37.0	[6.2, 430]	**< 0.001**				
		3	37.0	[6.2, 430]	**< 0.001**				
Invasion of	Capsular insertion	1	25.5	[3.7, + inf]	**< 0.001**	0.62	[0.45, 0.78]	0.94	[0.82, 1.00]
		2	9.3	[2.0, 62.0]	**0.002**				
		3	6.7	[1.6, 35.6]	**0.007**				
	Cruciate ligament	1	61.0	[7.2, + inf]	**< 0.001**	0.77	[0.55, 0.98]	0.81	[0.56, 1.00]
		2	10.7	[1.3, 148]	**0.02**				
		3	10.7	[1.3, 148]	**0.02**				
	Ligamentum teres	1	2.3	[0.2, + inf]	0.54	0.79	[0.48, 1.00]	1.00	[1.00, 1.00]
		2	4.6	[0.3, + inf]	0.26				
		3	4.6	[0.3, + inf]	0.26				

*Significant *p* values are printed bold. *OR*, odds radio; *95% CI*, 95% confidence interval; *ITT*, intrasynovial tumor tissue; *+inf*, upper limit of 95% confidence interval was infinite

**Table 4:** Performance of the analyzed imaging features in diagnosing JI

Imaging feature		Reader	Sensitivity (%, n, [95 % CI])			Specificity (%, n, [95 % CI])			PPV (%, n, 95 % CI)			NPV (%, n, 95 % CI])		
Tumor size > 10.5 cm		1	38	(9/24)	[18, 59)	63	(15/24)	[41, 81]	50	(9/18)	[32, 67]	50	(15/30)	[39, 61]
		2	29	(7/24)	[13, 51]	79	(19/24)	[58, 93]	58	(7/12)	[34, 79]	53	(19/36)	[45, 61]
		3	50	(12/24)	[31, 69]	63	(15/24)	[43, 79]	57	(12/21)	[37, 72]	56	(15/27)	[37, 72]
Epiphyseal/acetabular	Bone marrow replacement	1	96	(23/24)	[80, 100]	42	(10/24)	[22, 63]	62	(23/37)	[54, 70]	91	(10/11)	[58, 99]
		2	96	(23/24)	[80, 100]	46	(11/24)	[26, 67]	64	(23/26)	[55, 72]	92	(11/12)	[65, 99]
		3	96	(23/24)	[80, 99]	46	(11/24)	[28, 65]	64	(23/36)	[48, 78]	92	(11/12)	[65, 99]
	Edema-like signal intensity	1	33	(8/24)	[16, 55]	54	(13/24)	[33, 74]	42	(8/19)	[26, 60]	45	(13/29)	[34, 56]
		2	80	(19/24)	[58, 93]	42	(11/24)	[22, 63]	58	(19/33)	[48, 67]	67	(10/15)	[45, 83|
		3	83	(20/24)	[64, 93]	50	(12/24)	[31, 69]	63	(20/32)	[45, 77]	75	(12/16)	[51, 90]
Transarticular	Bone marrow replacement	1	4	(1/24)	[0, 21]	100	(24/24)	[85, 100]	100	(1/1)	[21, 100]	51	(24/47)	[39, 53]
		2	4	(1/24)	[0, 21]	100	(24/24)	[85, 100]	100	(1/1)	[21, 100]	51	(24/47)	[39, 53]
		3	8	(2/24)	[1, 27]	100	(24/24)	[86, 100]	100	(2/2)	[34, 100]	52	(24/46)	[49, 55]
	Edema-like signal intensity	1	8	(2/24)	[1, 27]	100	(24/24)	[86, 100]	100	(2/2)	[34, 100]	52	(24/46)	[49, 55]
		2	4	(1/24)	[0, 21]	100	(24/24)	[85, 100]	100	(1/1)	[21, 100]	51	(24/47)	[39, 53]
		3	4	(1/24)	[0, 21]	100	(24/24)	[85, 100]	100	(1/1)	[21, 100]	51	(24/47)	[39, 53]
Synovial contrast enhancement		1	96	(22/23)	[78, 100]	29	(7/24)	[12, 51]	56	(22/39)	[50, 63]	88	(7/8)	[48, 98]
		2	87	(20/23)	[66, 97]	54	(13/24)	[33, 74]	65	(20/31)	[53, 74]	81	(13/16)	[59, 93]
		3	87	(20/23)	[66, 97]	54	(13/24)	[33, 74]	65	(20/31)	[53, 74]	81	(13/16)	[59, 93]
Joint effusion		1	63	(15/24)	[41, 81]	75	(18/24)	[44, 81]	71	(15/21)	[53, 84]	67	(18/27)	[53, 81]
		2	54	(13/24)	[33, 74]	96	(23/24)	[79, 100]	93	(13/14)	[65, 99]	68	(23/34)	[57, 77]
		3	58	(14/24)	[39, 76]	92	(22/24)	[74, 98]	88	(14/16)	[64, 97]	69	(22/32)	[51, 82]
Direct visualization of ITT		1	92	(22/24)	[73, 99]	100	(24/24)	[86, 100]	100	(22/22)	[85, 100]	92	(24/26)	[76, 98]
		2	100	(24/24)	[86, 100]	92	(22/24)	[73, 99]	92	(24/26)	[76, 98]	100	(22/22)	[85, 100]
		3	96	(23/24)	[80, 100]	92	(22/24)	[74, 98]	92	(23/25)	[75, 98]	96	(22/23)	[80, 99]
Destruction of	Bone	1	96	(23/24)	[80, 100]	96	(23/24)	[79, 100]	96	(23/24)	[77, 99]	96	(23/24)	[77, 99]
		2	96	(23/24)	[80, 100]	79	(19/24)	[56, 93]	82	(23/28)	[68, 91]	95	(19/20)	[73, 99]
		3	100	(24/24)	[86, 100]	71	(17/24)	[51, 85]	77	(24/31)	[60, 89]	100	(17/17)	[82, 100]
	Cartilage	1	29	(7/24)	[13, 51]	100	(24/24)	[86, 100]	100	(7/7)	[65, 100]	59	(24/41)	[52, 65]
		2	79	(19/24)	[58, 93]	92	(22/24)	[73, 99]	90	(19/21)	[71, 97]	81	(22/27)	[67, 91]
		3	79	(19/24)	[58, 93]	92	(22/24)	[73, 99]	90	(19/21)	[71, 97]	81	(22/27)	[67, 91]
Invasion of	Capsular insertion	1	46	(11/24)	[28, 65]	100	(24/24)	[86, 100]	100	(11/11)	[74, 100]	65	(24/37)	[49, 78]
		2	58	(14/24)	[37, 78]	88	(21/24)	[68, 97]	82	(14/17)	[61, 93]	68	(21/31)	[56, 78]
		3	58	(14/24)	[37, 78]	83	(20/24)	[39, 76]	78	(14/18)	[55, 91]	67	(20/30)	[49, 81]
	Cruciate ligament	1	80	(8/10)	[44, 97]	100	(18/18)	[81, 100]	100	(8/8)	[68, 100]	90	(18/20)	[72, 97]
		2	60	(6/10)	[26, 88]	89	(16/18)	[65, 99]	75	(6/8)	[41, 93]	80	(16/20)	[65, 90]
		3	60	(6/10)	[26, 88]	89	(16/18)	[65, 99]	75	(6/8)	[41, 93]	80	(16/20)	[65, 90]
	Ligamentum teres	1	55	(5/11)	[23, 83]	100	(2/2)	[16, 100]	100	(6/6)	[34, 100]	29	(2/7)	[17, 43]
		2	73	(8/11)	[39, 94]	100	(2/2)	[16, 100]	100	(8/8)	[68, 100]	40	(2/5)	[20, 64]
		3	73	(8/11)	[39, 94]	100	(2/2)	[16, 100]	100	(8/8)	[68, 100]	40	(2/5)	[20, 64]

*PPV*, positive predictive value; *NPV*, negative predictive value; *95 % CI*, 95 % confidence interval; *ITT*, intrasynovial tumor tissue

### Direct signs of joint invasion

**Fig. 3:**
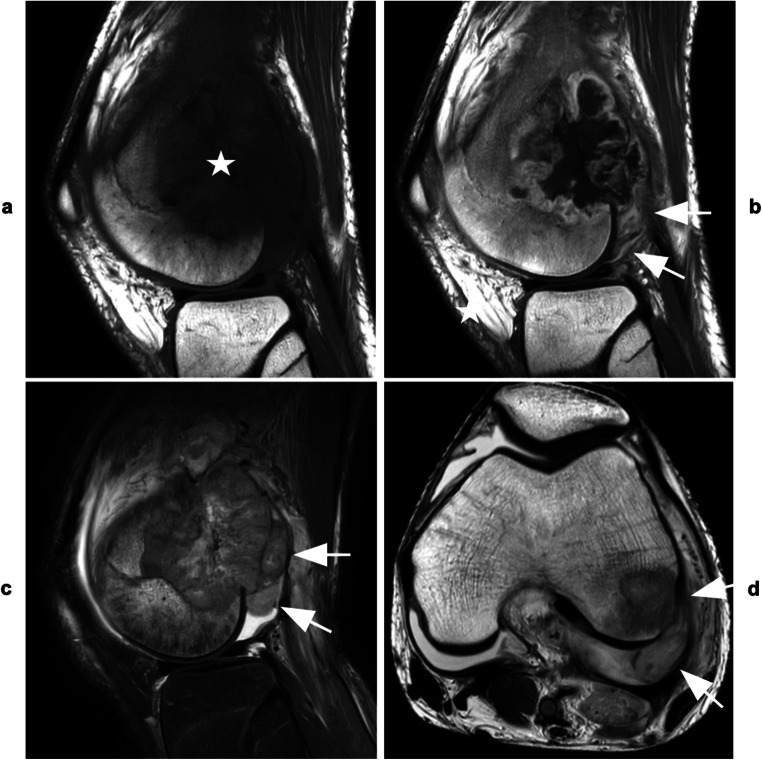
Osteosarcoma of the left distal femur invading the knee joint. **a** Sagittal T_1_-weighted TSE image shows metaphyseal mass (*star*) with epiphyseal extension and posterior soft tissue component. **b** Corresponding post-contrast image reveals inhomogeneous enhancement of the bone lesion as well as synovial enhancement at the posterior joint recess (*arrows*). **c** Sagittal intermediate-weighted TSE image with fat suppression and **d** axial T_2_-weighted TSE image show JI via the posterior capsular insertion and directly visualize intraarticular tumor tissue in contact with joint fluid and cartilage surface of the lateral femoral condyle (*arrows*)

**Fig. 4:**
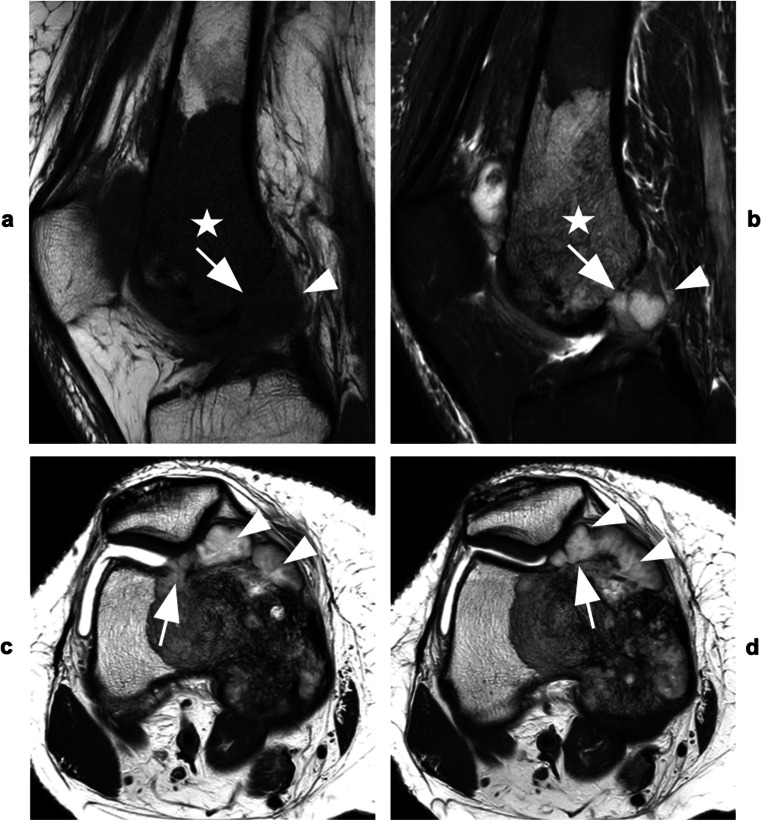
Chondrosarcoma of the right distal femur invading the knee joint at two different sites. **a** Sagittal T_1_-weighted and **b** corresponding intermediate-weighted TSE images with fat suppression demonstrate bone marrow replacement in the metaphysis and epiphysis of the distal femur (*stars*), destruction of intraarticular bone at the roof of the intercondylar notch (*arrows*), and tumor invasion of the anterior cruciate ligament (*arrowheads*). **c, d** Consecutive axial T_2_-weighted images show destruction of bone and articular cartilage at the femoral trochlea (*arrows*) as well as tumor nodules in contact with joint fluid and cartilage surface of the patella (*arrowheads*) indicating invasion of the femoro-patellar joint compartment

**Fig. 5:**
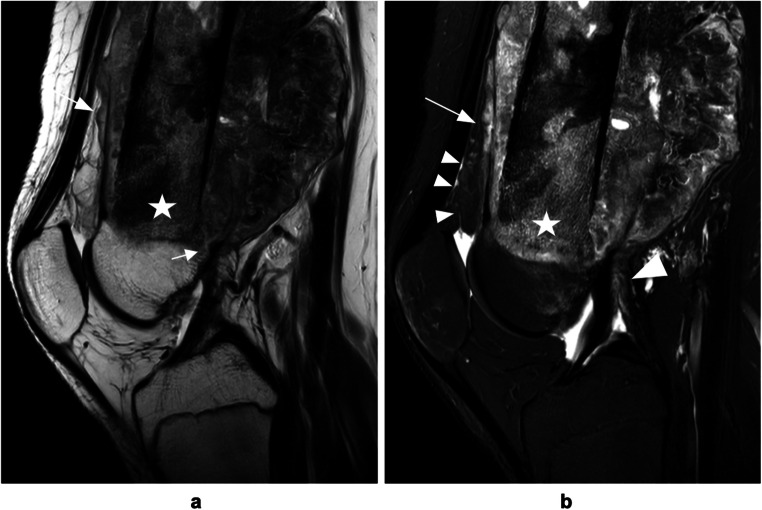
Extra-articular osteosarcoma of the right distal femur. **a** Sagittal T_2_-weighted TSE and **b** corresponding intermediate-weighted TSE images with fat suppression show inhomogeneous mass with large soft tissue component at the distal femoral diaphysis and metaphysis (*stars*). The tumor reaches the origin of the anterior cruciate ligament posteriorly (*short arrows*) but does not invade the ligament or cross the synovial membrane at the posterior capsule (*large arrowhead* in **b**). Anteriorly, the mass invades the prefemoral fat (*long arrows*) but does not reach the suprapatellar joint recess (*small arrowheads* in **b**)

All direct signs besides tumor invasion of the ligamentum teres were associated with increased odds for JI (Table 3). Direct visualization of ITT was strongly associated with the histopathological diagnosis of JI (OR [95 % CI], Reader 1 229 [29.8, + inf], *p* < 0.001; Reader 2 229 [29.8, + inf], *p* < 0.001; Reader 3 186 [17.8, 10848], *p* < 0.001) and associated values for sensitivity, specificity, PPV, and NPV were ≥ 92 % for all readers (Table 4; Figures 3 and 4). Moreover, inter- and intra-reader reproducibility were excellent (Fleiss’ *κ* [95% CI] = 0.83 [0.67, 1]; Cohen’s *κ* [95% CI] = 0.95 [0.86, 1.00]). Odds for JI were high in patients with intraarticular bone destruction (*p* < 0.001, respectively), and sensitivity of this sign as well as its NPV both ranged from 96 to 100%, while specificity (71–96%) and PPV (77–96%) were reader-dependent (Fleiss’ *κ* [95 % CI]; 0.72 [0.55, 0.88]). Destruction of cartilage showed elevated odds for JI in all readers (Reader 1 12.5 [1.7, +inf], 0.009; Readers 2+3 37.0 [6.2, 430], *p* < 0.001) and a specificity of 92 to 100%. With regard to intraarticular ligaments, analyses demonstrated increased odds for JI in cases affecting the cruciate ligaments, but not the ligamentum teres (*p* ≤ 0.02 and *p* ≥ 0.26, respectively). Capsular invasion predicted the histopathologic diagnosis of JI in all readers (*p* ≤ 0.007, respectively) and specificity was ≥ 83%, for each reader (Figure 5).

### Combinations of imaging features

Direct visualization of ITT and intraarticular destruction of bone met the criteria to be included in the MaxSens variable and MaxSpec comprised direct visualization of ITT, invasion of the capsular insertion, and cruciate ligaments or ligamentum teres. AllDirect had a sensitivity, specificity, PPV, and NPV of 96 % with one false-positive and one false-negative case (95 % CI; sensitivity, specificity: 79, 100; PPV, NPV: 77, 99). While results for MaxSens were the same, MaxSpec had a specificity and PPV of 100 % [86, 100], a sensitivity of 96 % [79, 100], and a NPV of 96 % [78, 99], with only one false-negative case.

## Discussion

This study evaluated the diagnostic performance and reproducibility of MR imaging features in the detection of JI by malignant bone tumors. Similar to previous population-based studies, osteosarcomas were the most frequent entity and the knee was the most common location for malignant bone neoplasms in our cohort [1]. Therefore, despite the relatively low number of included cases, we regard the external validity of our cohort as substantial.

Correct assessment of JI status in preoperative MR examinations is of great importance to orthopedic surgeons, as false-positive diagnoses lead to unnecessary extensive resections with high complication rates and worse functional outcomes, while false-negative diagnoses may increase the risk of local recurrence due to intra-operative spread of malignant cells across the surgical plane [5, 7, 8, 11, 18–20]. However, although previous studies found that the tumor diameter may precisely be measured using T_1_-weighted sequences, they also reported up to 50 % false-positive assessments, when JI was diagnosed with MR imaging [10, 12–14, 21]. In contrast, our readers were able to confidently exclude JI in 88–100 % of the true-negative cases. These differences may be explained by the improved quality of MR images compared to former years and the structured analysis of imaging signs in this study. This may also apply to the two most valuable and reproducible signs in this study—direct visualization of ITT and intraarticular destruction of bone—which were previously reported to be far less specific [12, 13, 22].

Previous studies have shown mixed results for the value of joint effusion and synovial contrast enhancement in diagnosing JI [12, 14]. Our results indicate that these indirect signs are either nonspecific or insensitive. Such reactive changes may occur due to hyperemia, capsular irritation by the inflammatory tumor microenvironment, or mechanical stress, if the tumor is abutting the joint, but not invading it [23]. Furthermore, the absence of joint effusion showed a much lower negative predictive value for JI than in the study by Schima et al (11).

Our readers were able to detect tumor invasion of capsular insertions and to differentiate this finding from mere capsular contact or displacement, in particular with use of T_2_-weighted images, whereas this task was described as challenging by Schima et al, and the usefulness of T_2_-weighted MR sequences for diagnosing JI has been discussed controversially [12, 24]. Tumor invasion of intraarticular ligaments was a highly specific sign and showed a high PPV. For the cruciate ligaments, these results match those of previous studies, while assessment of tumor invasion of the ligamentum teres has been reported to be more difficult, most likely due to technical limitations in visualizing the ligament [13]. Notably, invasion of the hip joint via the ligamentum teres occurred most often in patients with chondrosarcomas: Out of six cases, in which invasion of the hip joint was present, chondrosarcomas accounted for four.

Similar to previous reports, epiphyseal bone marrow replacement seen on T_1_-weighted MR images had an excellent sensitivity and NPV, but a low specificity for the diagnosis of JI in our study [12]. Edema-like epiphyseal signal alterations were however relatively insensitive and nonspecific and may thus only be of very limited value when diagnosing JI.

The combination of highly specific direct imaging features (direct visualization of ITT, invasion of the capsular insertion, or intraarticular ligaments) led to a further increase of sensitivity (96 %), while maintaining an excellent specificity (100 %). Consequently, we propose that a combination of direct signs should be considered when diagnosing JI.

This study has limitations: most importantly, a reader bias is pertinent to this study, as due to the design of the study, readers most likely expected a high number of cases with JI, possibly influencing the diagnosis. To minimize this bias, readers were blinded to clinical and histopathological diagnoses and surgical outcome [25]. Second, the analyzed examinations were obtained in clinical routine on two MR scanners with different field strengths using pulse sequences with different in-plane resolutions. On the other hand, this also demonstrates the feasibility of assessing the discussed imaging features in routine clinical scans. Third, the statistical power of our analyses is limited by the small cohort size. It may be difficult to overcome this limitation as the incidence of joint adjacent malignant bone tumors, particularly with JI, is relatively low.

In summary, our results demonstrate the feasibility to accurately determine JI status of malignant bone tumors on pre-therapeutic MR images. Direct signs, in particular visualization of intrasynovial tumor tissue and destruction of intraarticular bone, were found to be most valuable when assessing JI, owing to their excellent specificity, and were highly reproducible. In contrast to previous studies, indirect signs were found to be much less reliable. Providing this information is essential for orthopedic surgeons, as correctly diagnosing JI prior to surgery may prevent inadequate resections, postoperative complications, and local recurrence.

## Supplementary information

ESM 1 (DOCX 21 kb)

## Abbreviations

95% CI: 95% confidence interval
ITT: Intrasynovial tumor tissue
JI: Joint invasion
MR: Magnetic resonance
NPV: Negative predictive value
OR: Odds ratio
PPV: Positive predictive value
TSE: Turbo spin-echo

## Declarations

### Guarantor

The scientific guarantor of this publication is Prof. Dr. K. Woertler.

### Statistics and biometry

No complex statistical methods were necessary for this paper.

### Methodology

• retrospective

• diagnostic or prognostic study

• performed at one institution
