# Supplementary material for: Diagnosis of joint invasion in patients with malignant bone tumors: value and reproducibility of direct and indirect signs on MR imaging
Source: Eur Radiol. 2022 Mar 8;32(7):4738–48. doi: 10.1007/s00330-022-08586-w (PMC9213276; doi:10.1007/s00330-022-08586-w)
Supplement: Supplementary file 1 — (DOCX 21 kb) [file 330_2022_8586_MOESM1_ESM.docx]

| Supplementary Table: Magnetic Resonance imaging sequence parameters | | | | | | | | | | | | | | | | | | | | | | | | |
| --- | --- | --- | --- | --- | --- | --- | --- | --- | --- | --- | --- | --- | --- | --- | --- | --- | --- | --- | --- | --- | --- | --- | --- | --- |
|  | Knee | | | | | | | | Shoulder | | | | | | | | Hip / Pelvis | | | | | | | |
| Sequence | STIR | | T_1_ -/+ GD | | T_2_ | | T_1_-FS-GD | | IM-FS | STIR | T_1_ -/+ GD | | T_2_ | | T_1_-FS-GD | | STIR | | T_1_ -/+ GD | | T_2_ | | T_1_-FS-GD | |
| Field strength (T) | 1.5 | 3.0 | 1.5 | 3.0 | 1.5 | 3.0 | 1.5 | 3.0 | 1.5 | 3.0 | 1.5 | 3.0 | 1.5 | 3.0 | 1.5 | 3.0 | 1.5 | 3.0 | 1.5 | 3.0 | 1.5 | 3.0 | 1.5 | 3.0 |
| Repetition time (ms) | 4670 | 2850 | 557 | 1100 | 4020 | 4260 | 595 | 1050 | 3950 | 4500 | 537 | 1040 | 4300 | 4990 | 500 | 722 | 3470 | 9520 | 542 | 855 | 6110 | 5600 | 522 | 775 |
| Echo time (ms) | 69 | 47 | 15 | 12 | 104 | 94 | 14 | 13 | 45 | 47 | 16 | 12 | 89 | 78 | 16 | 12 | 31 | 44 | 15 | 12 | 85 | 87 | 13 | 12 |
| Flip angle (°) | 180 | 160 | 90 | 180 | 180 | 180 | 90 | 180 | 180 | 156 | 90 | 175 | 180 | 180 | 90 | 180 | 150 | 144 | 172 | 180 | 180 | 180 | 180 | 136 |
| Field of view (mm) | 220 | 180 | 220 | 200 | 160 | 160 | 160 | 160 | 160 | 180 | 160 | 160 | 180 | 180 | 160 | 180 | 260 | 350 | 260 | 350 | 180 | 350 | 180 | 350 |
| In-plane resolution (mm) | 0.7x 0.7 | 0.6x 0.6 | 0.5x 0.5 | 0.5x 0.5 | 0.5x 0.5 | 0.5x 0.5 | 0.6x 0.6 | 0.6x 0.6 | 0.2x 0.2 | 0.6x 0.6 | 0.2x 0.2 | 0.5x 0.4 | 0.4x 0.4 | 0.6x 0.6 | 0.3x 0.3 | 0.6x 0.6 | 0.8x 0.8 | 0.9x 0.9 | 0.4x 0.4 | 0.8x 0.7 | 0.6x 0.6 | 0.7x 0.7 | 0.6x 0.6 | 0.7x 0.7 |
| Slice thickness (mm) | 4 | 4 | 4 | 3 | 4 | 4 | 4 | 4 | 3 | 4 | 3 | 3 | 3 | 4 | 3 | 4 | 4 | 5 | 4 | 5 | 5 | 5 | 5 | 5 |
| Gap (%) | 50 | 25 | 25 | 20 | 25 | 10 | 25 | 10 | 10 | 25 | 10 | 10 | 20 | 20 | 20 | 20 | 60 | 40 | 20 | 50 | 60 | 40 | 60 | 40 |
| Bandwidth (Hz/pixel) | 130 | 252 | 90 | 172 | 111 | 200 | 94 | 150 | 109 | 252 | 85 | 161 | 109 | 200 | 85 | 150 | 161 | 246 | 128 | 160 | 119 | 203 | 130 | 160 |
| Echo train length (n) | 24 | 20 |  | 183 | 14 | 20 |  | 288 | 33 | 17 |  | 262 | 58 | 15 |  | 203 | 19 | 24 | 118 | 162 | 19 | 37 | 118 | 116 |
| Inversion time (ms) | 155 | 210 |  |  |  |  |  |  |  | 210 |  |  |  |  |  |  | 150 | 210 |  |  |  |  |  |  |
| STIR: short tau inversion recovery; GD: gadolinium-enhanced; FS: fat-saturated; IM: intermediate-weighted | | | | | | | | | | | | | | | | | | | | | | | | |
